# An optimized LSTM-based deep learning model for anomaly network intrusion detection

**DOI:** 10.1038/s41598-025-85248-z

**Published:** 2025-01-10

**Authors:** Nitu Dash, Sujata Chakravarty, Amiya Kumar Rath, Nimay Chandra Giri, Kareem M. AboRas, N. Gowtham

**Affiliations:** 1 Department of Computer Science and Engineering, BPUT, Rourkela, Odisha India; 2 Department of Computer Science and Engineering, Centurion University of Technology and Management, Bhubaneswar, Odisha India; 3 Department of Computer Science and Engineering, Faculty of Engineering, BPUT, Rourkela, Odisha India; 4 Department Electronics and Communication Engineering, Centurion University of Technology and Management, Jatni, Odisha 752050 India; 5 Department of Electrical Power and Machines, Faculty of Engineering, Alexandria University, 21544 Alexandria, Egypt; 6 Department of Electrical and Electronics Engineering, Manipal Institute of Technology Bengaluru, Manipal Academy of Higher Education, Manipal, India

**Keywords:** Intrusion detection system (IDS), JAYA optimization, Long short-term memory (LSTM), Particle swarm optimization (PSO), Salp swarm algorithm (SSA), Engineering, Mathematics and computing

## Abstract

The increasing prevalence of network connections is driving a continuous surge in the requirement for network security and safeguarding against cyberattacks. This has triggered the need to develop and implement intrusion detection systems (IDS), one of the key components of network perimeter aimed at thwarting and alleviating the issues presented by network invaders. Over time, intrusion detection systems have been instrumental in identifying network breaches and deviations. Several researchers have recommended the implementation of machine learning approaches in IDSs to counteract the menace posed by network intruders. Nevertheless, most previously recommended IDSs exhibit a notable false alarm rate. To mitigate this challenge, exploring deep learning methodologies emerges as a viable solution, leveraging their demonstrated efficacy across various domains. Hence, this article proposes an optimized Long Short-Term Memory (LSTM) for identifying anomalies in network traffic. The presented model uses three optimization methods, i.e., Particle Swarm Optimization (PSO), JAYA, and Salp Swarm Algorithm (SSA), to optimize the hyperparameters of LSTM. In this study, NSL KDD, CICIDS, and BoT-IoT datasets are taken into consideration. To evaluate the efficacy of the proposed model, several indicators of performance like Accuracy, Precision, Recall, F-score, True Positive Rate (TPR), False Positive Rate (FPR), and Receiver Operating Characteristic curve (ROC) have been chosen. A comparative analysis of PSO-LSTMIDS, JAYA-LSTMIDS, and SSA-LSTMIDS is conducted. The simulation results demonstrate that SSA-LSTMIDS surpasses all the models examined in this study across all three datasets.

## Introduction

In the modern age, substantial advancements have been made in the realm of Digital Technology, the Internet of Things (IoT), and diverse portable communication devices. The tremendous proliferation of these technologies has notably augmented the population and organizations depend on networks for executing a diverse range of tasks. The substantial rise in internet usage has led to a profound transformation in the lives of most individuals and the operational dynamics of numerous organizations. However, the swift expansion of internet services and extensive transmission of data have led to several security challenges in recent years. Security experts presented a variety of methods and concepts to address these challenges for securing networks^1^. Intrusion Detection Systems (IDSs) have been demonstrated to be highly effective in identifying and responding to network intruders. IDSs excel in both recognizing compromised network systems and detecting ongoing intrusion attempts. IDS monitors network traffic and conducts comprehensive network analysis. It detects potential threats as well as illegitimate network access^2^. IDS is software designed to gather and assess various security metrics associated with network. The term IDS encompasses three important methods: anomaly detection^3^, misuse detection^4^, and a fusion of both, referred as hybrid detection^5,6^. Anomaly detection signals an alert upon detecting a variation from a pre-defined network condition^7^. Anomaly detection excels at identifying behaviors that significantly diverge from the established patterns. Misuse detection involves evaluating attack patterns employed for system infiltration by cross-referencing them with recorded user activities. It’s crucial to recognize that intrusions can originate externally, known as outsider attacks, or internally, where authorized users seek heightened privileges, referred to as insider attacks.

Many researchers have suggested the application of machine learning (ML) techniques to detect and identify network intruders. The following are the most typical concerns with present machine learning-based solutions: Firstly, these models exhibit a notable false positive rate across a diverse spectrum of attacks^8^. Secondly, their lack of generalizability is apparent as existing studies predominantly rely on one dataset to assess the efficacy of machine learning models. Thirdly, the current models under scrutiny have failed to address the enormity of contemporary network traffic^9^. Lastly, there is an imperative need for solutions that can effectively navigate the escalating dimensions of today’s high-speed networks in terms of size, velocity, and dynamics. In contrast to classical machine learning, the contemporary paradigm of deep learning has showcased cutting-edge performance across various domains, notably excelling in challenges of intrusion detection^10,11^. It provides an improved representation of data, allowing for the design of better models. Long Short-Term Memory (LSTM) networks, a type of Recurrent Neural Network (RNN) has gained widespread adoption as a prominent algorithm in the realm of deep learning, particularly in the domain of intrusion detection classifications^12^.

The study is motivated by the challenges of machine learning techniques and the promising outcomes of deep learning. While LSTM networks have shown promise in sequence modeling tasks, their application in network intrusion detection face challenges such as computational complexity and resource requirements, difficulty in capturing long-term dependencies in network traffic patterns, suboptimal hyperparameter tuning, leading to reduced model performance. This research proposes an optimized LSTM-based deep learning model to address these challenges and enhance anomaly-based network intrusion detection systems’ accuracy, efficiency, and adaptability.

This article makes the following significant contribution:

- Development of an optimized LSTM model for network intrusion detection, leveraging PSO, JAYA, and SSA optimization techniques to enhance classification accuracy.
- Comprehensive evaluation using multiple benchmark datasets to ensure robust comparative analysis.
- Demonstrates the superior performance of SSA-LSTMIDS over PSO-LSTMIDS and JAYA-LSTMIDS showcasing improved accuracy, scalability, and efficiency across diverse datasets.

This paper is organized as: Section "Related works" provides a survey of machine learning and deep learning methodologies in the context of IDS classification. Section "Materials and Methods" outlines the Proposed framework and the methodologies employed for constructing the IDS model. This includes a comprehensive description of the Long Short-Term Memory (LSTM) model, as well as insights into the PSO, JAYA, SSA techniques. Section "Dataset description and pre-processing" details the NSL KDD, CICIDS 2017 & Bot-IoT dataset followed by data pre-processing steps. Section "Experimental results" elaborates the performance metrics and delineates the investigational outcomes along with their analysis, followed by the conclusion presented in Section "Conclusion and future scope".

## Related works

Machine learning algorithms employed in the field of intrusion detection have established themselves as highly effective approaches for securing network systems against unauthorized access. Many scholars in this field have presented a variety of machine learning algorithms. The researchers in^4,13,14^ have presented conventional machine learning methodologies for intrusion detection, including Naive Bayes (NB), Random Forest (RF), K-nearest neighbors (KNN) and Support Vector Machine. Despite yielding encouraging outcomes historically, these techniques possess inherent constraints, prompting the development of deep neural networks.

In a software-defined network^15^, the author used a deep learning technique to detect flow-based intrusions. The model demonstrated robust performance throughout the training and testing phases on the NSL-KDD dataset. A deep learning-based ID^16^ incorporated an LSTM framework within a Recurrent Neural Network (RNN) for training on the KDD Cup 1999 dataset. Various parameters were used for experiments to determine the best learning rate and size of hidden layer. The classifier’s effectiveness was measured and compared with existing IDS classifiers using tenfold cross-validation throughout the testing stage. The LSTM-RNN classifier showed the best detection rate, accuracy, and false alarm rate. Deep learning was found to be an excellent method for implementing IDSs. Spectral clustering and deep neural network algorithms were amalgamated^17^ for the identification of intrusion behaviors. To gain further insights and identify patterns from related clusters, the initial step involves collecting network features grouped by clusters, which were then partitioned into k subsets. In the second phase, deep learning networks were employed to extract highly abstract properties from the subsets generated during the clustering process. Finally, attacks were identified by testing these subsets. This methodology proved beneficial in improving detection rate accuracy. Researchers^18^ suggested a deep learning approach, for developing a classifier employing LSTM Recurrent Neural Network (LSTM RNN). Six different optimizers were investigated to discover the best optimizer among them. The LSTM RNN model, optimized with Nadam, proved to be the most effective intrusion detection technique, achieving a notable 97.54% accuracy, 98.95% high detection rate, and a relatively low 9.98% false alarm rate. Recurrent Neural Networks (RNN) serves as a deep learning model^19^ for identifying anomalies in network traffic data extracted from the NSL-KDD dataset. The authors validated that RNN outperformed conventional machine learning techniques, including J48, NB, RF and SVM. A recurrent neural network incorporating a gating mechanism i.e GRU-RNN^12^ identified intrusions within software-defined networks. NSL-KDD dataset was used for testing and evaluating their proposed method. They asserted that the implementation of GRU-RNN had no adverse effects on the performance of the network and enhanced the accuracy of anomaly detection. The intelligent network attack detection system introduced by LSTM-RNN^20^ consisted of three layers: input, mean pooling, and regression. The method demonstrated favorable results when applied to the NSL-KDD dataset. The presentation of a distinctive perspective on the aspect of information security was accomplished through the introduction of a multimodal sequential approach featuring a deep hierarchical progressive network, as outlined in reference^21^. LuNet, a deep neural network framework^22^ was designed for identifying attacks on a huge network. LuNet utilizes LSTM for capturing temporal features and CNN to extract spatial features from traffic data. The integration of both CNN and RNN is synchronized to investigate input data at the same granularity, aiming to prevent information loss. Additionally, batch normalization is incorporated in the design to enhance learning. Through experimental evaluations conducted on the NSL-KDD and UNSW-NB15 datasets, LuNet showcased enhancements in accuracy and a decrease in the false positive rate for network intrusion detection. These improvements positioned LuNet favorably compared to other state-of-the-art methods. A hybrid model^23^ combined CNN for feature extraction from large IDS data with a Weight Dropped LSTM (WDLSTM) network to preserve long-term dependencies across the obtained features, thereby preventing overfitting on recurrent connections. The UNSW-NB15 dataset was utilized to compare the suggested hybrid method with traditional approaches, demonstrating its satisfactory performance. In^24^, an ensemble IDS model is introduced, which integrates LR, NB and DT algorithms using a voting classifier to enhance detection performance. The model is evaluated on the CICIDS2017 dataset, demonstrating robust results with an accuracy of 88.96% in multiclass classification and 88.92% in binary classification. This approach highlights the effectiveness of combining multiple algorithms through ensemble methods to improve overall detection accuracy across different classification tasks.In another research^25^, a comprehensive model for identifying and categorizing network attacks using recurrent deep learning architectures is presented. The suggested model extracts hidden layer features from recurrent models and additionally applies a kernel-based principal component analysis (KPCA) technique for optimal feature selection. Subsequently, the optimal features derived from recurrent models are amalgamated, and classification is executed through an ensemble meta-classifier. Furthermore, a new CFAEE-SCA algorithm, designed to improve the basic Firefly Algorithm (FA), was developed with enhancements like gBest chaotic local search (CLS) strategy and hybridization with sine–cosine algorithm (SCA) to optimize the XGBoost classifier for intrusion detection^26^. In^27^ intrusion detection is enhanced by using a Transformer model with an improved position encoding method that better captures feature dependencies, along with using a stacked auto-encoder for dimensionality reduction, applying a hybrid data sampling method combining KNN-based under-sampling and Borderline-SMOTE to balance the dataset leading to higher classification accuracy. It achieves improved performance on the NSL-KDD dataset over UNSW-15 dataset with faster training compared to existing models. Optimizing LightGBM for IDS using the Grasshopper Optimization Algorithm (GOA) presents a promising approach to enhance detection accuracy and efficiency^28^. This methodology leverages the strengths of LightGBM, a gradient boosting framework, combined with GOA for hyperparameter tuning, achieving a detection rate of approximately 97% on the UNSW-NB15 dataset. This study^29^ utilizes particle swarm optimization and genetic algorithm (PSO-GA) for feature selection on the CICIDS-2017 dataset and proposes a hybrid model, GRU-LSTM, combining gated recurrent unit and long short-term memory for improved accuracy in intrusion detection. Achieving 98.86% accuracy, the proposed model outperforms current methods, as validated through a comparative study, demonstrating its efficacy in detecting various network attacks. An improved LSTM method integrated with RNN i.e. ILSTM-RNN is introduced to bolster security in IDS^30^. The devised system addresses the gradient-clipping problem using likely point particle swarm optimization (LPPSO) and refined LSTM classification. Findings indicate that the suggested IDS-centric model exhibits a superior detection rate compared to alternative machine learning (ML) and RNN-centric methodologies. An in-depth comparison of feature extraction and feature selection methods for intrusion detection in IoT networks, focusing on a machine learning-based attack classification framework is presented in^31^. DT, RF, KNN, NB, MLP are the different ML techniques used. It evaluates key performance metrics such as accuracy, F1-score, and runtime using the TON-IoT dataset for both binary and multiclass classification tasks. Feature extraction yielded better performance with smaller feature sets, whereas feature selection excelled in reducing training and inference times. Another study implements six classification approaches ie Extreme Gradient Boosting (XGBoost), RF, Bidirectional Auto-Regressive Transformers (BART), Logistic Regression (LR), Multivariate Adaptive Regression Splines (MARS), DT and a stacked ensemble model for attack classification in IoT scenarios^32^. To improve performance, six sampling techniques (undersampling, oversampling, ROSE, SMOTE, b-SMOTE, ADASYN) and dimensionality reduction (PCA, PLS) were applied. A novel scoring system was introduced to select the best models based on accuracy and execution time. The ensemble method demonstrated a substantial improvement in accuracy performance.

## Materials and methods

### Proposed framework

The objective of the proposed IDS model is to construct a dependable computational intelligence model capable of precisely identifying malicious network activities. To develop the model four steps are involved.

**Fig. 1:**
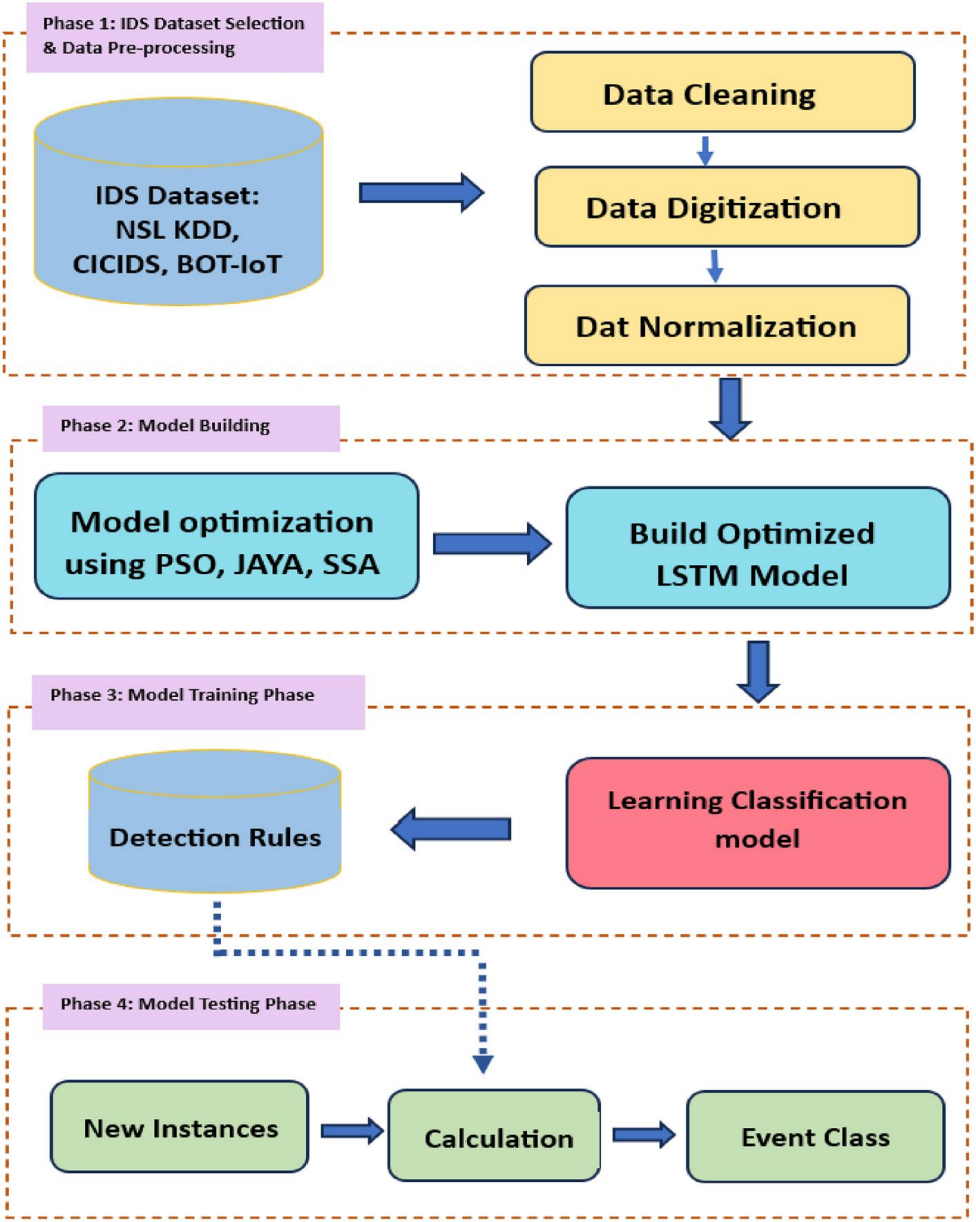
Proposed framework of optimized LSTMIDS.

First, NSL KDD, CICIDS & Bot-IoT IDS dataset are selected for experimentation followed by data preprocessing which includes Data cleaning, data digitization and normalization. Secondly, LSTM is used to build the IDS model. PSO, JAYA and SSA optimization techniques are used to fine-tune the hyperparameters of the LSTM system for enhancing the classification performance. Thirdly, the training dataset is employed to train the optimized LSTM framework. The system undergoes training using records comprising both normal and attack data, facilitating the storage of detection rules. Finally, testing dataset is applied to assess the performance of the optimized LSTM model, ensuring its ability to generalize well to new data through the application of detection rules. Figure 1 illustrates the different phases of the proposed framework.

### Long short-term memory (LSTM)

**Fig. 2:**
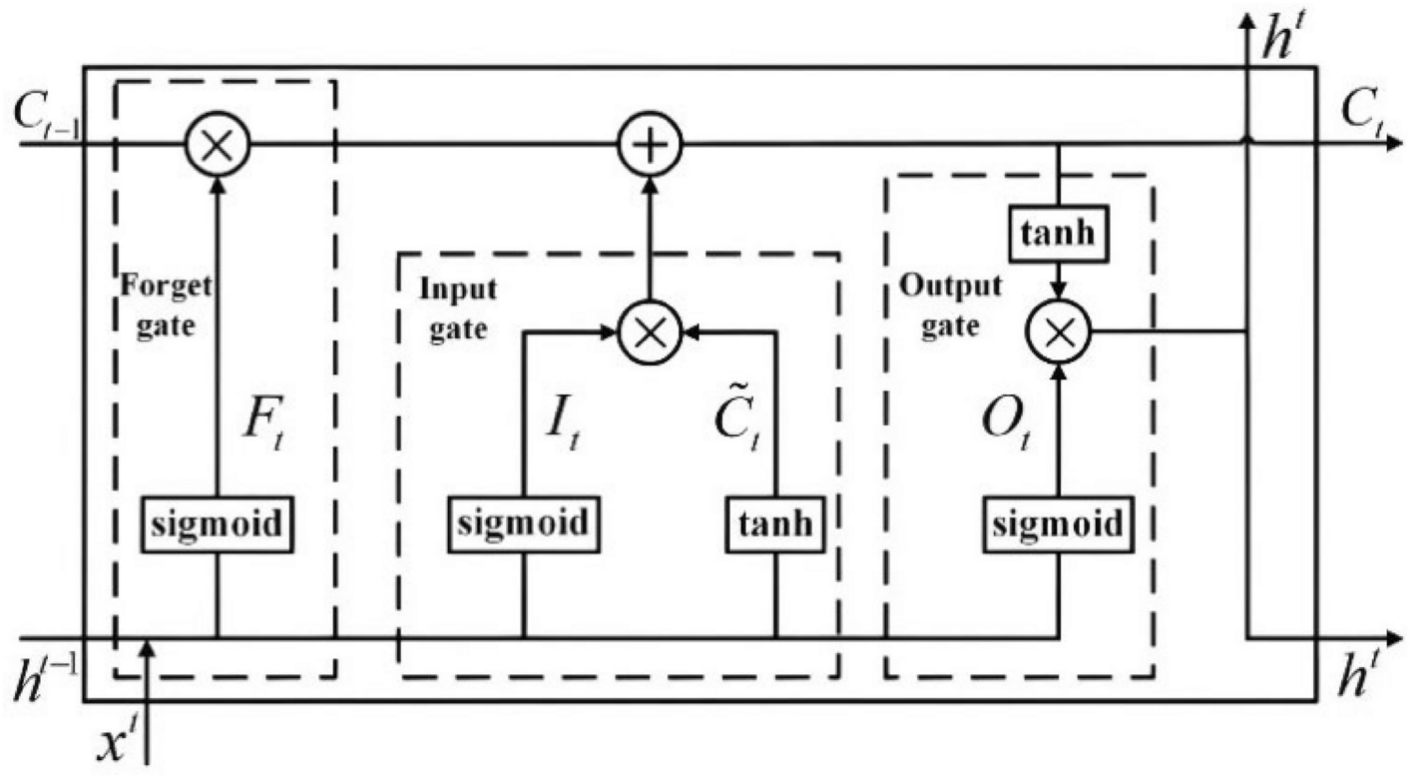
Cell structure of LSTM.

LSTM networks are a specialized form of Recurrent Neural Networks (RNNs) designed to address the vanishing gradient problem inherent in traditional RNNs, enabling the efficient learning of long-term dependencies^33^. By incorporating a gating mechanism, LSTMs allow for better management of information over time, facilitating the retention and forgetfulness of data as required. The LSTM architecture comprises cell states and three essential gates: input, forget, and output gates. The cell state functions as a conveyor belt, transporting crucial information through the network^34^. Input gates regulate the addition of new information to the cell state, forget gates filter out irrelevant data from previous states, and output gates determine the information from the cell state that contributes to the hidden state and final output. This gating system enables LSTMs to effectively manage the flow of information, ensuring relevant data is retained and utilized while extraneous information is discarded. Thus, LSTMs excel in tasks requiring accurate temporal awareness and long-term dependency learning. The cell structure of LSTM is given below in Fig. 2.

The following equations depict the relationship between inputs and outputs at time *t* and *t* – 1 :

1 $$i_{t} = \sigma (W_{{xi}} \chi _{t} + W_{{hi}} h_{{t - 1}} + W_{{ci}} c_{{t - 1}} + b_{i} )$$

2 $$f_{t} = \sigma (W_{xf} \chi_{t} + W_{hf} h_{{t - {1}}} + W_{{cf\;ct - {1}}} + b_{f} )$$

3 $$c_{t} = f_{t} c_{t - 1} + i_{t} tanh(W_{xc} \chi_{t} + W_{hc} h_{t - 1} + b_{c} )$$

4 $$o_{t} = \sigma (W_{xo} \chi_{t} + W_{ho} h_{t - 1} + W_{co} c_{t - 1} + b_{o} )$$

5 $$h_{t} = o_{t} tanh(c_{t} )$$

where *c* represents the cell state. σ and tanh represents the activation functions. *x* denotes the input vector, ℎ_*t*-1_ represents the hidden state from the previous timestamp. *W* and *b* denotes the weights and bias parameters. *f*_*t*_ is the forget function and filters out irrelevant information. *i*_*t*_ is the input gate and *c* introduce new information to the cell state. *o*^*t*^ is the output gate responsible for producing the pertinent information as output.

### Particle swarm optimization (PSO)

Drawing insights from observations of bird migration and eating habits, Kennedy and Eberhart^35^ explored the possibility of optimizing nonlinear functions applying a particle swarm technique. In this method, each solution within the search dimension is denoted as a particle, characterized by position, velocity, and fitness value. The particle’s position represents a potential solution within the search space, its velocity dictates both direction and distance, and its fitness value is calculated by the objective function, reflecting the quality of the solution. The PSO algorithm’s formulas for updating velocity and position are as follows.

6 $${V}_{i,d}^{k+1 }={\omega v}_{i,d}^{k}+{c}_{1}{r}_{1}({pbest}_{i,d}^{k}- {x}_{i,d}^{k})+{c}_{2}{r}_{2}({gbest}_{d}^{k}- {x}_{i,d}^{k})$$

7 $${x}_{i,d}^{k+1}= {x}_{i,d}^{k}+ {v}_{i,d}^{k+1}$$

where ${v}_{i,d}^{k+1}$ signifies the velocity of particle *i* in the *d-*dimension at *k*^th^ iteration; ${\omega v}_{i,d}^{k}$ represents the inertia, i.e., the tendency of the particle to continue moving in its current direction with its previous velocity; ${c}_{1}{r}_{1}({pbest}_{i,d}^{k}- {x}_{i,d}^{k})$ reflects the cognitive component, where the particle is attracted towards its personal best position; ${c}_{2}{r}_{2}({gbest}_{d}^{k}- {x}_{i,d}^{k})$ represents the social component, where the particle is attracted towards the best position found by any particle in the swarm.

In this study, an LSTM classification model is developed and the key parameters in the LSTM are fine-tuned using the PSO algorithm. The finalized model is applied to IDS dataset.

### Jaya algorithm

Jaya is a gradient-free optimization algorithm capable of addressing both constrained and unconstrained optimization problems^36^. This method operates as a population-based approach, iteratively modifying a population of individual solutions. It does not contain any hyperparameters.

Three steps make up the Jaya algorithm: initialization, updation of feasible solutions, and greedy selection. Each of the CS feasible solutions presented by the initial colony displays D dimension variables^37^. The initialization optimization problem can provide feasible solutions as follows:

8 $${x}_{j,m}= {x}_{j,m }^{l} {+ rand(x}_{j,m}^{u}- {x}_{j,m}^{l}),$$

where ${x}_{j,m}$ signifies the value of mth variable of the jth feasible solution; ${x}_{j,m}^{u}$ and ${x}_{j,m}^{l}$ denotes the upper and lower bounds of ${x}_{j,m}$; and rand signifies a random number in the interval [0,1]. The feasible solutions undergo modifications. ${x}_{j,m}^{(g)}$ signifies the modified value in the gth iteration which can be calculated as

9 $$x_{{j,m}}^{{\left( g \right)\prime }} = x_{{j,m}}^{{\left( g \right)}} + rand\left( {x_{{best,m}}^{{\left( g \right)}} - \left| {x_{{j,m}}^{{\left( g \right)}} } \right.} \right) - rand\left( {x_{{worst,m}}^{{\left( g \right)}} - \left| {x_{{j,m}}^{{\left( g \right)}} } \right.} \right)$$

where ${x}_{best,m}^{\left(g\right)}$ and ${x}_{worst,m}^{\left(g\right)}$ represents the best value and worst value of the mth variable in gth iteration. rand$({x}_{best,m}^{\left(g\right)}$–ǀ ${x}_{j,m}^{\left(g\right)})$ refers to the inclination of the best solution to feasible solutions. rand$({x}_{worst,m}^{\left(g\right)}$- ǀ ${x}_{j,m}^{\left(g\right)})$ refers to the inclination of feasible solutions to avoid from the worst one. Subsequently, the below specified boundary condition is applied.

10 $${{x}_{j,m}^{\left(g\right)}}{\prime}= \left\{\begin{array}{c}{x}_{j,m}^{\left(l\right)} if {{x}_{j,m}^{\left(g\right)}}{\prime}< {x}_{j,m}^{\left(l\right)} \\ {x}_{j,m}^{u} if {x}_{j,m}^{\left(g\right){\prime}}> {x}_{j,m}^{u}\\ {x}_{j,m}^{{\left(g\right)}{\prime} } otherwise\end{array}\right.$$

By including all dimensions in the updating rule and boundary judgement, a new feasible solution ${{x}_{j}^{\left(g\right)}}^{{{\prime}}}$ will be produced. The greedy selection criterion is employed to evaluate if the recently generated feasible solution ${{x}_{j}^{\left(g\right)}}^{{{\prime}}}$ or the prior one ${x}_{j}^{\left(g\right)}$ will survive in the subsequent iteration. It is anticipated that a viable solution with a lower objective function value will endure in the subsequent iteration. The iterative procedure persists until the maximum iteration number is reached.

To enhance the classification accuracy of the LSTM model and achieve optimal results, hyperparameters undergo tuning through the Jaya optimization algorithm. This choice is made due to its simplicity in application and its effective optimization performance. The JAYA algorithm efficiently converges to global optimum values with a reduced number of iterations and computational time.

### Salp swarm algorithm (SSA)

Mirjalili et al.^38^ introduced a heuristic group optimization method known as the Salp Swarm Algorithm (SSA) in the year 2017. The SSA algorithm seeks out the optimal parameters by imitating the swarming mechanism of salps when foraging in oceans. The salp group in the sea is organized in a chain like structure; the foremost salp leads the entire swarm, and the subsequent salps scan the environment in a forward direction. The SSA’s procedure is given below:

- Population initialization. SSA uses random number generation to initialize the population.
  11 $${\text{X}}_{{{\text{M }} \times {\text{ d}}}} = {\text{lb}} + {\text{rand}}\left( {{\text{M}},{\text{d}}} \right) \times \left( {{\text{ub}} - {\text{lb}}} \right)$$
  **M** signifies the count of salps, **I** signify the highest iteration limit and **[lb, ub]** represents the search spectrum. The parade target’s dimensions are given by **d**.
- Compute each salp’s fitness. Store the salp’s coordinate with the best fitness.
- Determine variable c_1_.
  12 $${\text{c}}_{{1}} = {\text{2e}}^{{ - ({\text{ 4i}}/{\text{I}})}}$$
  where I signify the present iteration count; and I signifies the highest number of iteration.
- Update the position of first salp. The first directs the migration of the salp population toward food. According to the updated equation, the initial salp position is:
  13 $${x}_{d}^{1}=\left\{\begin{array}{c}{P}_{d}+ {c}_{1}\left(\left({ub}_{d}-{lb}_{d}\right){c}_{2}+{lb}_{d}\right),{c}_{3}\ge 0.5\\ {P}_{d}- {c}_{1}\left(\left({ub}_{d}-{lb}_{d}\right){c}_{2}+{lb}_{d}\right),{c}_{3}<0.5\end{array}\right.$$
  where, ${\text{x}}_{\text{d}}^{1}$ represents the leading salp position within a d dimensional space; ub_d_ and lb_d_ denotes the upper and lower bounds. P_d_ refers to the food source position within the same d dimensional space; c_2_ and c_3_ represent random values uniformly generated within the range [0, 1].
- Update the follower’s position by applying equation
  14 $${x}_{d}^{m}= \frac{1}{2}[{x}_{d}^{m}+{x}_{d}^{m-1}]$$
  where, m-1, ${x}_{d}^{m}$ denotes the positional parameters of the mth Salp in the space of d dimension.
- Determine each salp’s fitness.
- Save the coordinates of Salp with the best fitness. Update i = I + 1
- If i > I, output the salp coordinates with the best fitness; else, proceed to step 3.

Fusion of Salp Swarm Algorithm (SSA) with LSTM is an approach that integrates a nature-inspired optimization algorithm with a deep learning model for IDS classification. SSA-LSTMIDS is a sophisticated approach to IDS classification, leveraging attention mechanisms and LSTM to effectively model and classify network traffic data.

**Algorithm: LSTM for IDS Classification with PSO, JAYA, and SSA for Hyperparameter Tuning**

**Step 1: Data Preprocessing**

- ***Load Data*** *: Load the intrusion detection dataset (NSL-KDD, CICIDS2017, BoT-IoT).*
- ***Data Cleaning*** *: Handle missing values, normalize features.*
- ***Split Data*** *: Split the dataset into training and testing sets.*

**Step 2: Define LSTM Model**

- ***Function Definition:*** *Create an LSTM model function that takes hyperparameters as inputs. These hyperparameters include the number of LSTM units, dropout rate, number of layers, learning rate, and optimizer type.*
- ***Model Construction:*** *Within the LSTM function, construct the model with the specified hyperparameters.*

**Step 3: Define hyperparameters and search space**

- ***Hyperparameters*** *: Define a list of hyperparameters to optimize (e.g., number of LSTM units, learning rate, batch size, number of epochs).*
- ***Search Space*** *: Define the range or possible values for each hyperparameter.*

**Step 4: Implement optimization algorithms (PSO, JAYA, SSA)**

**PSO (Particle swarm optimization):**

- *Initialize a population (swarm) of particles with random positions and velocities in the search space.*
- *Evaluate the fitness of each particle using the validation accuracy of the LSTM model.*
- *Update particles’ velocities and positions based on personal best and global best positions.*
- *Iterate until convergence criteria are met (maximum iterations or convergence threshold).*

**JAYA algorithm:**

- *Initialize a random population.*
- *Evaluate the fitness (validation accuracy) of each solution.*
- *For each iteration, move solutions toward the current best solution while moving them away from the worst solution.*
- *Update the population and iterate until convergence criteria are met.*

**SSA (Salp swarm algorithm):**

- *Initialize a population of salps (solutions).*
- *Evaluate the fitness (validation accuracy) of each salp.*
- *Update the positions of the salps based on a combination of exploration and exploitation strategies.*
- *Iterate until convergence criteria are met.*

**Step 5: Train and evaluate LSTM model**

- ***Best hyperparameters*** *: Select the best hyperparameters obtained from the optimization step.*
- ***Train LSTM model*** *: Train the LSTM model on the training data using the best hyperparameters.*
- ***Evaluate model*** *: Evaluate the LSTM model on the testing data and calculate metrics such as accuracy, precision, recall, F1-score.*

## Dataset description and pre-processing

### Datasets

In this study, NSL-KDD, CICIDS2017 and BoT-IoT datasets are taken into consideration. These benchmark datasets are chosen for their diversity and representation of different network environments and attack types. These datasets offer a balanced mix of legacy attacks (NSL-KDD) to real-world traffic patterns (CICIDS) and IoT-specific threats (BoT-IoT), providing a robust foundation for evaluating IDS models.

### NSL-KDD dataset

Tavallaee^39^ designed the NSL-KDD dataset, represents an improved downsized subset of the KDD 99 dataset and has gained recognition for evaluating Intrusion Detection System (IDS) methodologies. The dataset consists of a total of 42 features out of which 41 represents the characteristic features of the data and 1 represents the type of attack. The training dataset incorporates 21 distinct attacks, while the testing dataset contains 37 diverse attack types. These attacks are classified into four groups: Denial of Service (DoS), Probe, User to Root (U2R), and Remote to Local (R2L), as detailed in Table 1. The NSL-KDD dataset is one of the datasets used for testing in the current study.

**Table 1:** Attack types of NSL KDD dataset.

Category	Attacks type (37)
DoS	Apache2, Land, Pod, Process table, Mailbomb Neptune, Teardrop, Back, Edstrom, Smurf, Worm
Probe	Nmap, Satan, IPsweep, Saint, Portsweep, Mscan
R2L	Multihop Guess_password, Xlock, Phf, Named, imap, Snmpgetattack, Sendmail, Ftp_write, Httptunnel, Snmpguess, Waremaster, Xsnoop
U2R	Rootkit, Perl, Ps, Xterm, Loadmodule, Sqiattack, Buffer_overflow

The normal and attack traffic distribution is outlined in Table 2.

**Table 2:** NSL KDD dataset distribution of normal and attack traffic.

Dataset	Normal	DoS	Probe	R2L	U2R	Total
KDDTrain +	67,343	45,927	11,656	995	52	**125,973**
KDDTest +	9710	7458	2422	2887	67	**22,544**

Significant values are in bold.

This dataset has multiple versions, and for this experiment, 20% of the training data is utilized and identified as "KDDTrain + _20Percent," comprising a total of 125,192 instances. Similarly, the test dataset “KDDTest + " contains a total of 22,544 instances.

### CICIDS2017 dataset

The CICIDS2017 dataset^40^, introduced by the Canadian Institute for Cybersecurity (CIC) in late 2017, encompasses detailed labels for timestamp data, source and destination IP addresses, source and destination ports, protocols, and various types of attacks. The dataset used the Email, FTP, SSH, HTTP, and HTTPS protocols to generate the abstract behavior of 25 users. Various time periods are used to collect the data. The attacks in this dataset are classified into six categories: DoS, PortScan, Bot, Brute Force, Web Attacks and Infiltration. The B-Profile system is employed for profiling abstract facets of human interactions, while the Alpha profile is utilized to simulate diverse multi-stage attack scenarios.

The dataset encompasses attack information derived from five days of traffic data. In CSV format, the dataset comprises a total of 2,830,743 rows distributed across 8 separate files, and each row is characterized by 79 features. Every entry is classified as either “Benign” or linked to one of fourteen distinct types of attacks. Additionally, it was discovered that the dataset has 203 instances of missing information and 288,602 instances of missing class labels. The merged dataset of CICIDS2017 contains 2,830,540 occurrences after these missing cases are removed.

To generate training and test subset, all rows where "Flow Packets/s" feature is either ‘Infinity’ or ‘NaN’ are deleted. Subsequently, the identical rows are deleted. After elimination, the training and testing subsets are extracted as outlined in Table 3. The aim is to incorporate rows encompassing all the attacks while ensuring no two rows are identical in any of the subsets. For the training subset, the initial rows corresponding to each attack type are opted. Subsequently, for the testing subset, additional rows after eliminating those rows already included in the training subset are randomly selected.

**Table 3:** Normal and attack traffic distribution of CICIDS2017 dataset.

Category	Attack Type	Total	Total (-Rows with lack Info)	Training	Test
BENIGN	BENIGN	2,273,097	2,271,320	50,000	50,000
DOS	DDoS	128,027	128,025	17,924	20,484
	DoS slow loris	5796	5796	1159	1449
	DoSSlowhttptest	5499	5499	825	1100
	DoS Hulk	231,073	230,124	20,711	25,314
	DoSGoldenEye	10,293	10,293	721	926
	Heartbleed	11	11	1	1
PortScan	PortScan	158,930	158,804	11,116	14,292
Bot	Bot	1966	1956	137	176
Brute-Force	FTP-Patator	7938	7935	555	952
	SSH-Patator	5897	5897	413	531
Web attack	Web Attack- Brute Force	1507	1507	161	176
	Web Attack-XSS	652	652	88	102
	Web Attack-SQL Injection	21	21	6	8
Infiltration	Infiltration	36	36	9	13
Total attacks		471,454	470,365	53,825	65,524
Total		2,830,743	2,827,876	103,825	115,524

An additional noteworthy discovery is that the dataset fulfills all the criteria outlined in references^41^ for a real intrusion detection dataset. This includes comprehensive network configuration, labeled dataset with traffic information, interactive capture, support for various protocols, diverse attack scenarios, system heterogeneity, a well-defined feature set, and associated metadata. For our experiment, 103,825 training records and 115,524 testing records from the ICIDS-2017 dataset have been utilized.

### BOT-IOT dataset

Bot-IoT dataset, published in 2019 by Koroniotis et al.^42^ originates from the Cyber Range Lab of UNSW Canberra. Distinguished as the first Intrusion Detection System (IDS) dataset of its kind, the Bot-IoT testbed incorporates IoT-generated network traffic and presents realistic Botnet scenarios. The testbed structural design is composed of three principal elements: the network platform, simulated IoT services, and the extraction of features and forensic analytics. Traffic generation is facilitated by the Ostinato tool, while simulation is achieved using the Node-red tool.

The Bot-IoT consists of numerous sets and subsets with various file types, sizes, and feature counts. The dataset comprises four distinct sets: the raw set, the full set, the 5% subset, and the 10-Best subset. The raw set includes approximately 70 GB of binary packet capture files (PCAP). via network taps in the testbed environment. The Argus tool is used by the Bot-IoT authors to create their processed dataset. The Full Set is the first processed set of data which consists of several CSV files. It is split into training and testing set which includes 9543 normal traffic and 73,360,900 malicious traffic. The Full Set comprises 3 dependent features and 26 independent features consisting of the network flow metadata from Argus while excluding the 14 computed features suggested by Koroniotis et al.^42^. The full set lacks header rows in its CSV files and contains six columns dispersed across features with empty data. These details are vital for researchers when importing or converting the data for analysis. Bot-IoT comprises two separate subsets that are frequently employed in academic studies. Koroniotis et al. offered and suggested a scaled-down 5% Subset as a more compact and easily handled version of the dataset. It roughly includes 3.6 million instances having 43 independent features and 3 dependent features with a header row. The 10-Best Subset^43^, incorporates only 10 features with 3 dependent features and 6 independent features. The selection of the 10-Best features is accomplished by mapping the correlation coefficient and joint entropy of the 43 independent features within the 5% Subset. Features are chosen according to their high scores in both measures, ensuring their superior performance. This subset is divided into two CSV files, each with a header row listing feature names such as pkSeqID, Seq, Mean, Stddev, Min, Max, Srate, Drate, NINConn, PSrcIP, and PDstIP. The training and test datasets are structured with 5 output classes, each representing either normal traffic or any four types of cyberattacks, namely Information Gathering, Denial of Service, Distributed Denial of Service, and Information theft. Table 4 outlines 5% of the entire training and testing dataset, specifying that 107,634 instances are used for training and 112,842 instances for testing in our experiment conducted with the Bot-IoT dataset.

**Table 4:** Normal and attack traffic distribution of Bot-IoT dataset.

Category	Attack type	Total count	Training	Test
BENIGN	BENIGN	9543	7634	1909
Information gathering	Service scanning	1,463,364	20,306	22,267
	OS Fingerprinting	358,275	6231	7166
DDoS attack	DDoS TCP	19,547,603	21,355	23,176
	DDoS UDP	18,965,106	16,543	18,128
	DDoS HTTP	19,771	470	650
DoS attack	DoS TCP	12,315,997	13,000	14,200
	DoS UDP	20,659,491	21,228	24,237
	DoSHTTP	29,706	640	760
Information theft	Keylogging	1469	185	294
	Data theft	118	42	55
Total	*–*	73,370,443	107,634	112,842

### Data preprocessing

The primary goal of data preprocessing in IDS datasets is to clean and transform the data to make it suitable for training and testing anomaly detection models^32^. Preprocessing datasets for IDS classification improves model performance by enhancing data quality, reducing noise, and addressing issues like missing or imbalanced data. It also boosts the model’s generalizability by making it more resilient to diverse data distributions and unseen attack types.

**Step-1 Data cleaning:** Dataset includes missing values and duplicate records. Duplicate records are removed. Missing values are handled by imputing values or removing incomplete records.

**Step-2 Data digitization:** The dataset incorporates a combination of character and numeric valued attributes. Character valued attributes in the dataset are converted to numeric value.

**Step-3 Data normalization:** Normalizing the dataset within the range of [0–1] is performed to enhance classification accuracy. High value range features are linearly scaled to the range [0.0 to 1.0] using Eq. 15. This scaling process helps standardize and normalize the feature values for consistency in the dataset.

15 $$f=\frac{f-min}{max -min}$$

where *f* = feature value, *max* = maximum value, *min* = minimum value of the feature.

## Experimental results

All the experiments detailed in this paper are conducted using a computing system equipped with Intel(R) Core (TM) i7-9750H processor running at 2.60 GHz. All the three models are implemented using the Keras 2.13.1 framework within a Jupyter Notebook environment utilizing Python 3.9. Performance indicators for IDS classification are derived from four potential aspects^44^:

*True Positive (TP):* attacks correctly labeled as intrusions.

*True Negative (TN):* normal activities correctly labeled as normal.

*False Positive (FP)*: normal activities erroneously labeled as intrusive.

*False Negative (FN):* attacks erroneously labeled as normal.

The following metrics, namely Accuracy (ACC), Precision, and Recall, are computed based on the above criteria:

Accuracy (ACC) determines the overall correctness of the IDS, calculated as the ratio of accurately classified instances (both True Positives and True Negatives) to the total number of instances.

16 $$Accuracy \left(ACC\right)=\frac{(TP+TN)}{(TP+TN+FP+FN)}$$

Recall or True Positive Rate (TPR) is computed as the percentage of accurately predicted attacks out of the total count of attacks.

17 $$Recall or TPR=\frac{TP}{(TP+FN)}$$

False Positive Rate (FPR) determines the percentage of genuine normal instances incorrectly classified as attack.

18 $$FPR=\frac{FP}{(FP+TN)}$$

Precision measures the ratio of correctly identified attack activities among all instances classified as attacks by the IDS.

19 $$Precision=\frac{TP}{(TP+FP)}$$

F-score (F1) is evaluated by combining precision and recall, presenting a balanced indicator of a model’s efficiency in a classification task.

20 $$F1=2*\frac{(Precision * Recall)}{(Precision + Recall)}$$

Receiver Operating Characteristics (ROC) is a graphical depiction of binary classification model’s effectiveness, illustrating the trade-off between true positive rate (sensitivity) and false positive rate (1—specificity) at various classification thresholds.

21 $$ROC\, = \,TPR \, vs \, \left( {1 - \, TNR} \right)$$

This study introduces an optimized deep-learning model designed for IDS classification. The proposed framework dynamically chooses optimal training parameters and establishes the execution cycle utilizing the optimization techniques. The experiments are carried out on three different datasets ie NSL KDD, CICIDS 2017 and BoT-IoT. This experiment uses only 20% of the data from each NSL KDD dataset, 10,3825 training records and 115,524 testing records from the ICIDS-2017 dataset, Similarly, 107,634 training instances and 112,842 testing instances from the Bot-IoT dataset has been utilized.

The initial parameter setup in the LSTM neural network plays a crucial role in determining the network’s efficiency. Three different optimization techniques i.e. PSO, JAYA and SSA are utilized to fine-tune the hyperparameters of LSTM and the models are compared.

The LSTM model employs a sequential architecture with Keras and TensorFlow. The preprocessing involves reshaping the input datasets to add an additional dimension, transforming the data into a shape compatible with Conv1D layers. A fixed random seed (7) ensures reproducibility. The first layer is a 1D convolutional layer (Conv1D) with 30 filters and a kernel size of 5, using ‘same’ padding to retain input dimensions and Rectified Linear Unit (ReLU) activation for non-linearity. This is followed by a max pooling layer (MaxPooling1D) with a pool size of 2 for dimensionality reduction. An LSTM layer with 120 units and a 0.2 dropout rate captures temporal dependencies, preventing overfitting. A Dense layer with 512 neurons and sigmoid activation is added for binary classification. The model is compiled with the binary_crossentropy loss function and adam optimizer, tracking accuracy. It is trained for 300 epochs with a batch size of 120. Evaluation on test data, with suppressed output verbosity (verbose = 0), provides the model’s accuracy. Table 5 outlines the parameters and hyper parameters of LSTM for classification.

**Table 5:** LSTM model parameters and hyperparameters for classification.

Layer type	Name	Configuration
Input	Input layer	Input features of pp1 and pp2 reshaped to (number_of_samples, sequence_length, 1)
Hidden	Conv1D	Filters = 30, Kernel Size = 5, Padding = ‘same’, Activation = ReLU
	MaxPooling1D	Pool Size = 2
	LSTM	Neuron Units = 120, Dropout rate = 0.2
	Dense	Neurons = 512, Activation = Sigmoid
Output	Output layer	Binary classification output using sigmoid activation
Hyperparameters		Early Stopping (monitor = ‘loss’, verbose = 1, patience = 6)
		Optimizers = Adam
		Loss Function = binary_crossentropy
		Learning Rate = 0.001
		Batch Size = 120
		Epochs = 300
Evaluation	Model evaluation	Verbose = 0, Accuracy metric printed as "%.2f%%" of (scores[1]*100)

The PSO algorithm is employed to optimize the hyperparameters of an LSTM-based neural network model designed for binary classification. The hyperparameters tuned using PSO are the number of LSTM units and the number of filters in the Conv1D layer. The lower and upper bounds for these hyperparameters are set to [50, 10] and [150, 50], respectively, representing the search space dimensions. The PSO algorithm is configured with a population size of 1 and is run for 1000 iterations. Key PSO parameters included a maximum velocity (Vmax) of 6, inertia weights (wMax and wMin) ranging from 0.9 to 0.2, and cognitive (c1) and social (c2) coefficients both set to 2. The objective function involved training the LSTM model with hyperparameters specified by each particle’s position and using 1—accuracy on the test set as the fitness metric to be minimized. The PSO algorithm iteratively updated the particles’ positions and velocities to explore the search space effectively, converging on the optimal hyperparameters for the LSTM model.

Similarly, JAYA and SSA algorithm is utilized to tune the hyperparameters of an LSTM model focusing on the number of LSTM units and Conv1D filters. The lower and upper bounds for the LSTM units are defined between 50 and 150, while for Conv1D filters, the bounds are set between 10 and 50. The dim (dimension) is set to 30, indicating the number of hyperparameters being optimized. SearchAgents_no is 1, meaning only one search agent is used. Max_iter is set to 1000, which specifies the maximum number of iterations the algorithm will perform to find the optimal hyperparameter values. This optimization process aims to find the best hyperparameters that minimize the objective function’s value, improving the model’s performance in binary classification task.

Time, Training accuracy (TrgAcc), Testing accuracy (TstAcc), Precision, Recall and F-score of PSO-LSTMIDS, JAYA-LSTMIDS and SSA-LSTM model are depicted from Tables 6, 7 and 8. Table 6 presents the evaluation results of the PSO-LSTMIDS model on the NSL KDD, CICIDS 2017, and Bot-IoT datasets, employing filters between 10 and 30. For NSL KDD, the model’s performance enhances with an increase in filters, achieving a peak training accuracy of 94.67%, testing accuracy of 91.05%, and an F-score of 90.71% at 30 filters. The CICIDS 2017 dataset exhibits marked improvements, with testing accuracy reaching 94.69% and precision at 98.46% with 30 filters, albeit with an increased computation time of 26.27 s. The Bot-IoT dataset shows moderate gains, attaining a testing accuracy of 83.89% and an F-score of 80.00% at 30 filters, alongside a rise in computational time. Overall, additional filters tend to bolster performance across all datasets. Table 7 presents dataset performance of JAYA-LSTMIDS model. Notably, with 30 filters: NSL KDD achieves high accuracy with 96.93% training and 95.14% testing accuracy, CICIDS 2017 excels in precision at 99.17%, and BoT-IoT achieves strong recall at 88.18%. Across all datasets, F-score improves with more filters. However, computational time increases as more filters are employed, with BOT-IOT requiring the most time at 26.94 s. Similarly, Table 8 compares SSA-LSTMIDS performance metrics across all the three datasets with various filter counts. With 30 filters, NSL KDD achieves impressive testing accuracy of 97.89%, while CICIDS 2017 reaches 99.80% and BOT-IOT attains the highest accuracy of 98.80%. Precision, recall, and F-Score also improve with more filters, showcasing the models’ ability to balance accuracy metrics.

**Table 6:** PSO-LSTMIDS training & testing accuracy, precision, recall, F-Score on NSL KDD, CICIDS & Bot-IoT dataset.

Dataset	NSL KDD					CICIDS 2017					BOT-IOT				
No. of Filters	10	15	20	25	30	10	15	20	25	30	10	15	20	25	30
Time (sec)	10.29	13.14	15.82	16.49	18.33	12.24	16.36	18.44	22.98	26.27	14.36	19.21	19.65	23.22	26.11
TrgAcc	0.91	0.9106	0.9211	0.9219	0.9467	0.9168	0.9357	0.9511	0.9528	0.9691	0.82	0.8201	0.8432	0.8436	0.8588
TstAcc	0.8762	0.86	0.8891	0.9021	0.9105	0.9011	0.9032	0.9098	0.9247	0.9469	0.8102	0.8106	0.8324	0.8326	0.8389
Precision	0.8409	0.8409	0.8421	0.8466	0.8533	0.9628	0.9684	0.9701	0.9811	0.9846	0.7411	0.741	0.7584	0.7621	0.7697
Recall	0.9106	0.9211	0.9301	0.9302	0.9333	0.8435	0.8422	0.8456	0.8437	0.8667	0.8136	0.8201	0.8302	0.8338	0.8463
F-Score	0.8821	0.8841	0.8868	0.8934	0.9071	0.8106	0.8211	0.8234	0.8269	0.8387	0.7814	0.7819	0.782	0.7901	0.800

**Table 7:** JAYA- LSTMIDS training & testing accuracy, precision, recall, F-Score on NSL KDD, CICIDS & Bot-IoT dataset.

Dataset	NSL KDD					CICIDS 2017					BOT-IOT				
No. of Filters	10	15	20	25	30	10	15	20	25	30	10	15	20	25	30
Time (sec)	10.36	12.64	13.58	19.44	21.21	13.21	13.24	19.22	21.47	26.94	10.25	12.22	12.59	18.25	22.34
TrgAcc	0.9358	0.9344	0.9489	0.9506	0.9693	0.9492	0.9511	0.9662	0.9801	0.9889	0.501	0.8521	0.8711	0.8711	0.8903
TstAcc	0.9206	0.92	0.9321	0.9411	0.9514	0.9301	0.9421	0.9602	0.9711	0.9714	0.8105	0.8215	0.8254	0.8302	0.8425
Precision	0.8821	0.8811	0.8902	0.8957	0.9068	0.9732	0.9811	0.9825	0.9883	0.9917	0.7384	0.7516	0.7574	0.7698	0.7857
Recall	0.9768	0.9768	0.9824	0.9855	1	0.9147	0.9251	0.9304	0.9442	0.9431	0.8305	0.8431	0.8687	0.8754	0.8818
F-Score	0.9402	0.9411	0.9415	0.9506	0.953	0.9024	0.9111	0.9154	0.922	0.9261	0.8158	0.8205	0.8255	0.8319	0.8429

**Table 8:** SSA-LSTMIDS training & testing accuracy, precision, recall, F-Score on NSL KDD, CICIDS & Bot-IoT dataset.

Dataset	NSL KDD					CICIDS 2017					BOT-IOT				
No. of Filters	10	15	20	25	30	10	15	20	25	30	10	15	20	25	30
Time (sec)	11.21	12.14	12.16	15.94	19.32	10.11	10.16	14.98	17.25	20.33	10.2	12.54	17.91	18.26	19.32
TrgAcc	0.9765	0.9754	0.9811	0.9884	0.9991	0.9736	0.9711	0.9754	0.9894	0.9992	0.9168	0.9211	0.9225	0.9387	0.9388
TstAcc	0.9753	0.9761	0.9788	0.9798	0.9789	0.9658	0.9639	0.9699	0.9706	0.9980	0.8169	0.8259	0.8467	0.8649	0.8644
Precision	0.8965	0.8954	0.8944	0.9021	0.9167	0.9792	0.9754	0.9798	0.9834	1	0.7592	0.7599	0.7842	0.8027	0.8021
Recall	0.9544	0.9424	0.9467	0.9592	1	0.9312	0.9311	0.9348	0.9471	0.9444	0.7936	0.7999	0.8149	0.886	0.8928
F-Score	0.9511	0.9463	0.9598	0.9602	0.973	0.9301	0.9312	0.9346	0.9469	0.9444	0.8314	0.8311	0.8425	0.859	0.8551

It is observed that SSA-LSTMIDS consistently demonstrates the highest testing accuracy across all datasets compared to PSO-LSTMIDS and JAYA-LSTMIDS. For the NSL KDD dataset, SSA-LSTMIDS achieves a testing accuracy range of 97.53–97.98%, significantly outperforming PSO-LSTMIDS (86–91.05%) and JAYA-LSTMIDS (92–95.14%). Similar trends are observed in the CICIDS 2017 and Bot-IoT datasets, with SSA-LSTMIDS achieving 96.39–99.80% and 81.69–86.49%, respectively. In terms of computational time, SSA-LSTMIDS generally shows faster or comparable times, especially for the CICIDS 2017 dataset, where it ranges from 10.11 to 20.33 s, demonstrating efficiency alongside high accuracy.

**Fig. 3:**
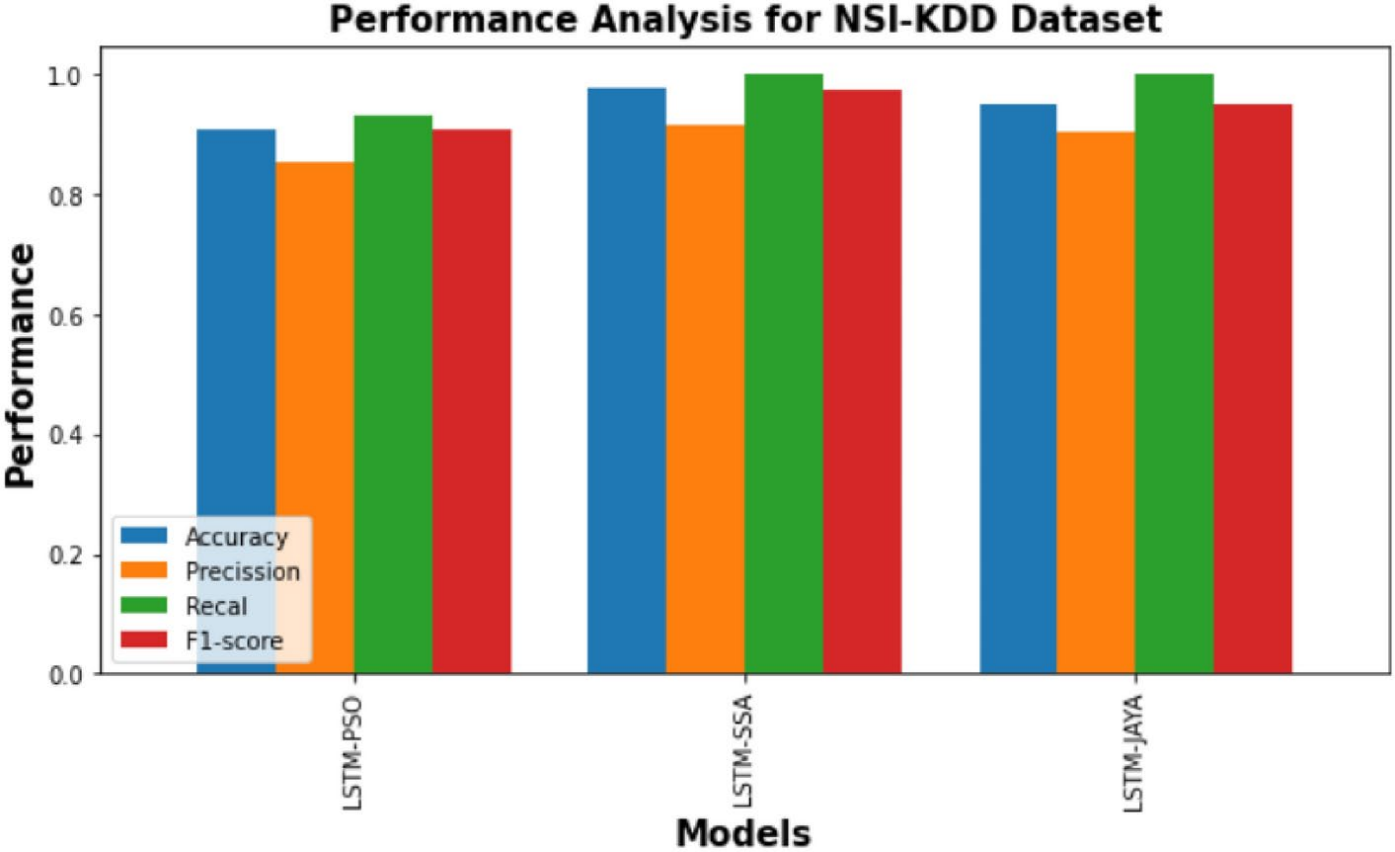
Performance comparison of PSO-LSTMIDS, JAYA-LSTMIDS, SSA-LSTMIDS on NSL KDD dataset. The bar chart compares across four metrics: Accuracy (blue), Precision (orange), Recall (green), and F1-score (red). Accuracy measures overall correctness, Precision quantifies the proportion of correct positive identifications, Recall reflects the correct identification of actual positives, and the F1-score balances Precision and Recall.

**Fig. 4:**
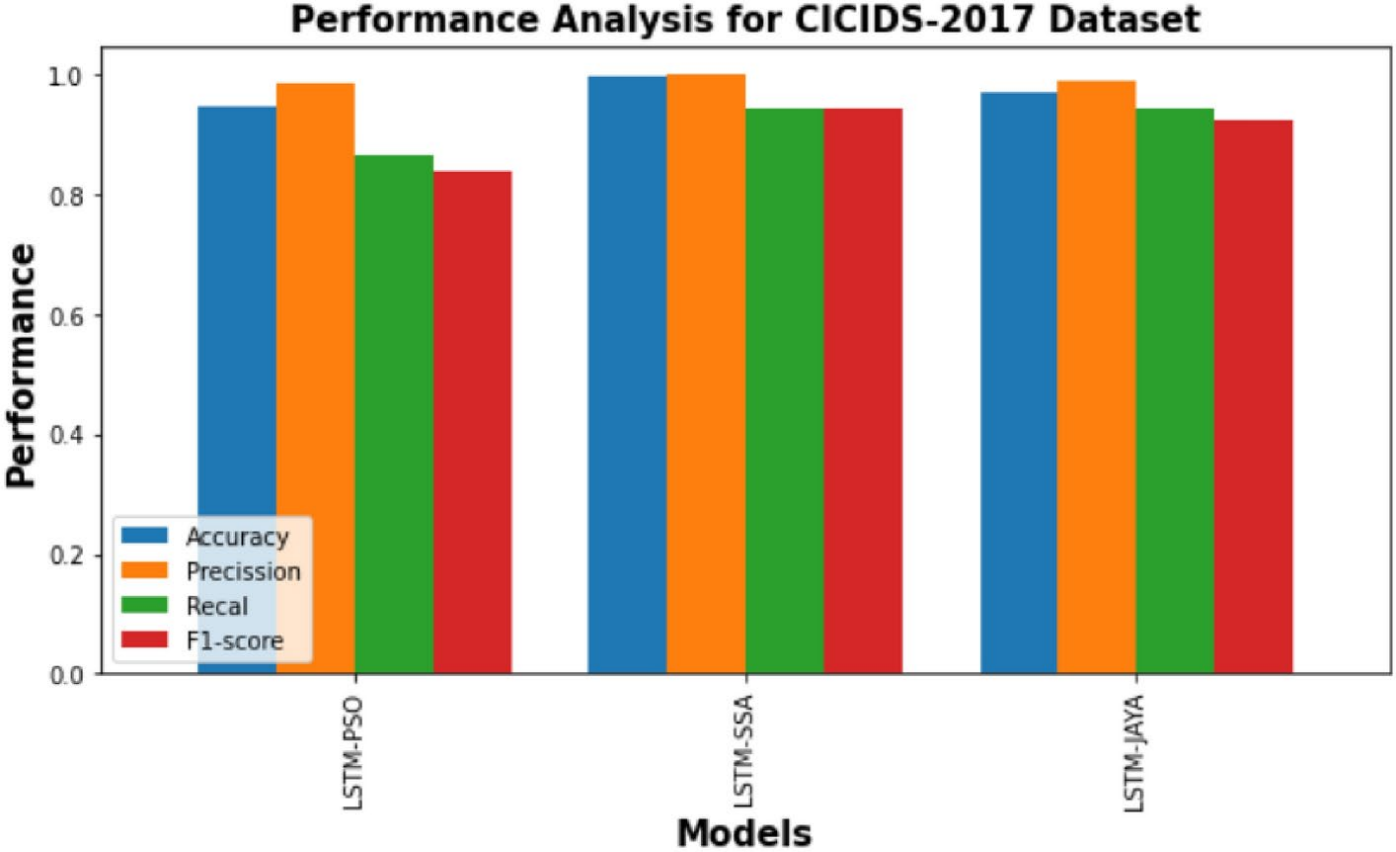
Performance comparison of PSO-LSTMIDS, JAYA-LSTMIDS, SSA-LSTMIDS on CICIDS 2017 dataset. Each bar in the figure represents the corresponding metric’s value for the given model. The height of the bar indicates the performance level, with higher values indicating better performance.

**Fig. 5:**
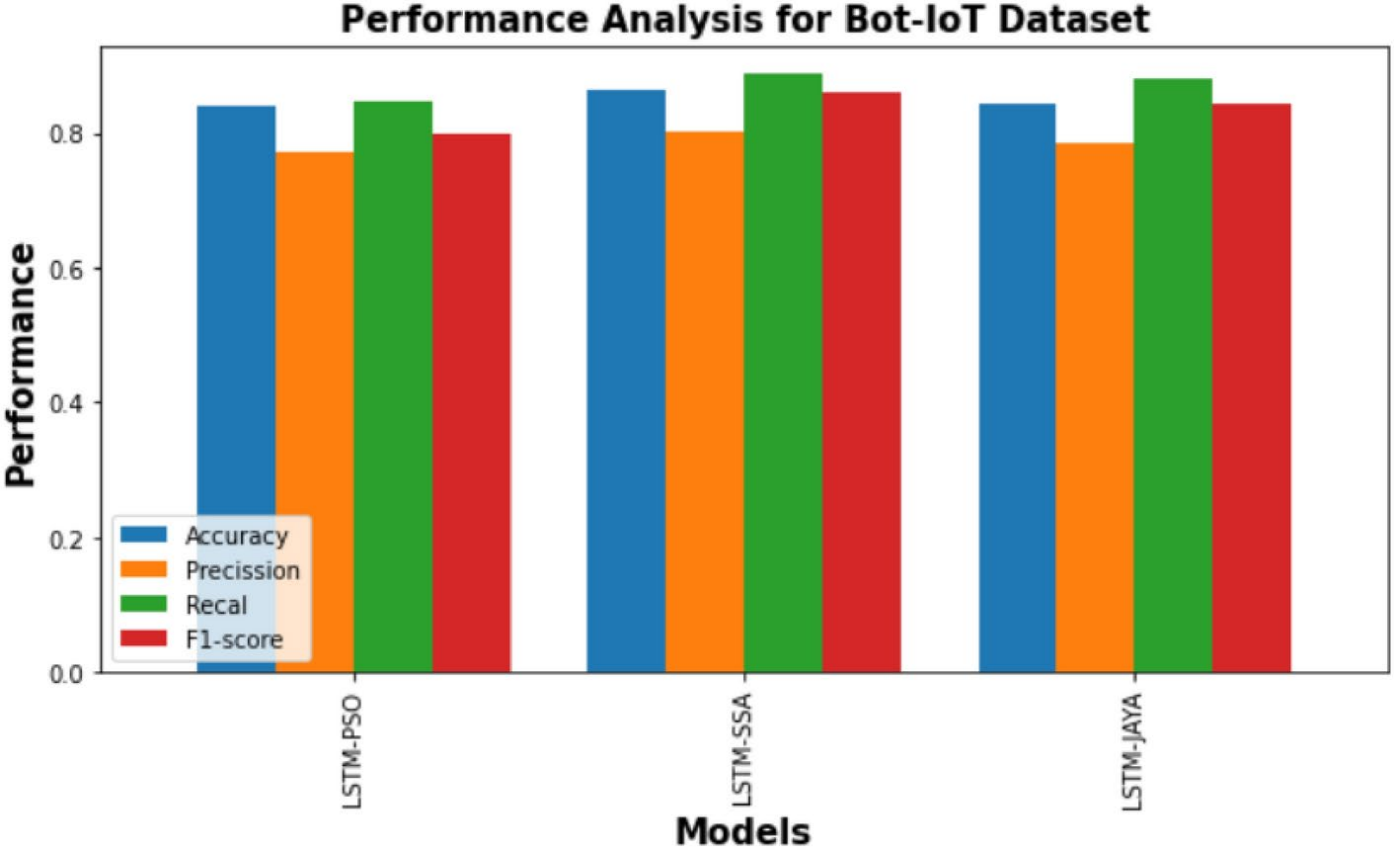
Performance comparison of PSO-LSTMIDS, JAYA-LSTMIDS, SSA-LSTMIDS on Bot-IoT dataset. The y-axis shows the performance values, ranging from 0 to 1. The x-axis lists the three models being compared. The chart indicates that the performance of the models is measured in terms of the four metrics ie accuracy, precision, recall, F1Score and the height of each bar corresponds to the value of the respective metric for each model.

Figure 3 is a bar graph illustrating the performance of three models— LSTM-PSO, LSTM-SSA, and LSTM-JAYA on the NSL-KDD dataset. Similarly Figs. 4 and 5 provides a comparison of the performance of LSTM-PSO, LSTM-SSA, and LSTM-JAYA models on the CICIDS 2017 and Bot-IoT dataset. Each model is assessed using four metrics: Accuracy, Precision, Recall, and F1-score. PSO-LSTM and JAYA-LSTM exhibit marginally higher precision and recall, while SSA-LSTM shows balanced performance across all metrics.

**Fig. 6:**
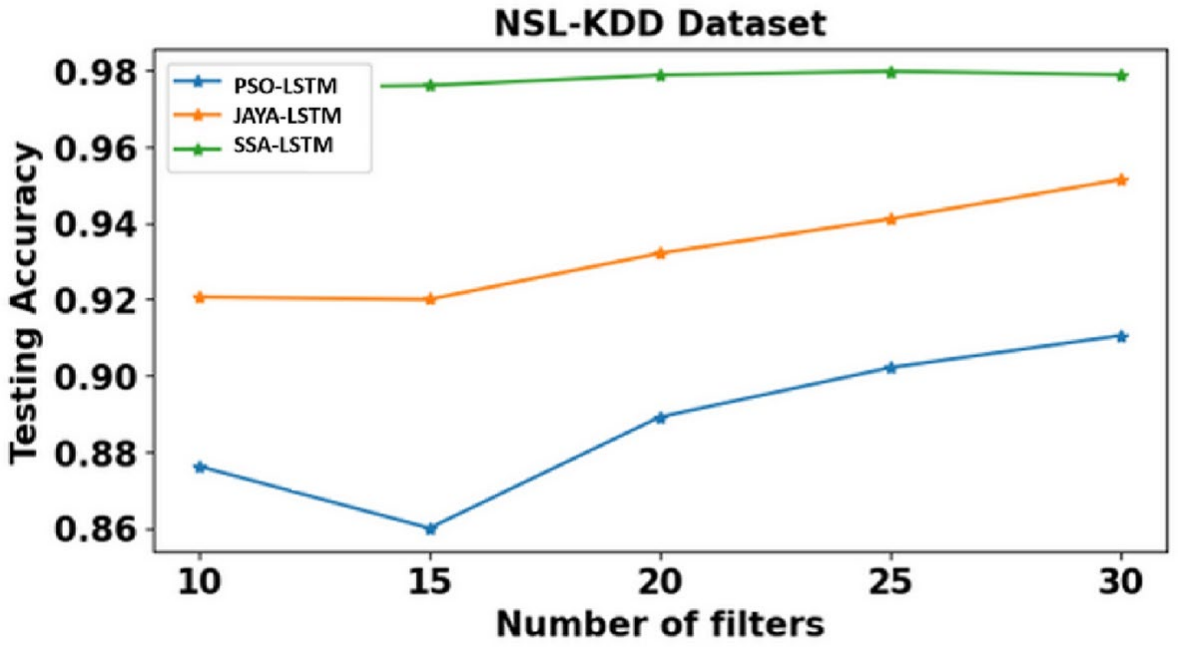
Testing accuracy comparison. The line graph titled "NSL-KDD Dataset" illustrates the testing accuracy of three different LSTM models—PSO-LSTMIDS, JAYA-LSTMIDS, and SSA-LSTMIDS—across a range of filter numbers from 10 to 30. The x-axis represents the number of filters, while the y-axis shows testing accuracy from 0.86 to 0.98. The data indicates that all models improve in accuracy as the number of filters increases. SSA-LSTMIDS, represented by a green line with square markers, consistently achieves the highest testing accuracy. JAYA-LSTMIDS follows, marked by an orange line with triangular markers, and PSO-LSTMIDS, denoted by a blue line with circular markers, shows the lowest but steadily improving accuracy.

**Fig. 7:**
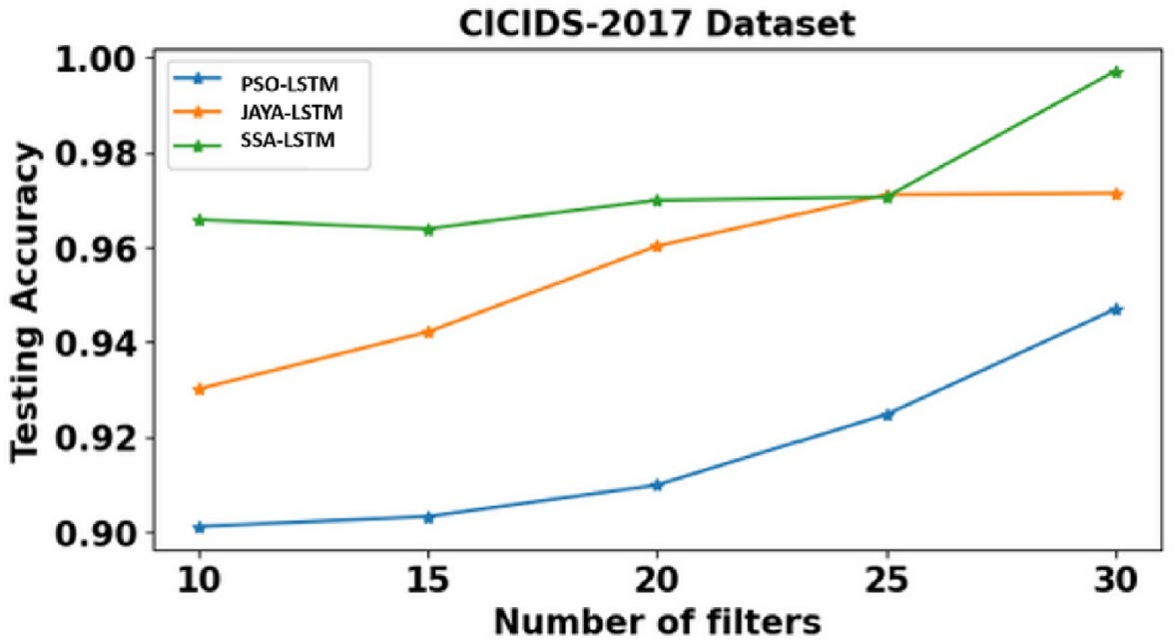
Testing accuracy comparison. The “CICIDS 2017” graph shows testing accuracy for PSO-LSTMIDS, JAYA-LSTMIDS, and SSA-LSTMIDs with filters from 10 to 30. Accuracy, on the y-axis (0.86 to 0.98), improves as filters increase. SSA-LSTMIDS (green squares) has the highest accuracy, JAYA-LSTMIDS (orange triangles) is mid-range, and PSO-LSTMIDS (blue circles) has the lowest but rises steadily.

**Fig. 8:**
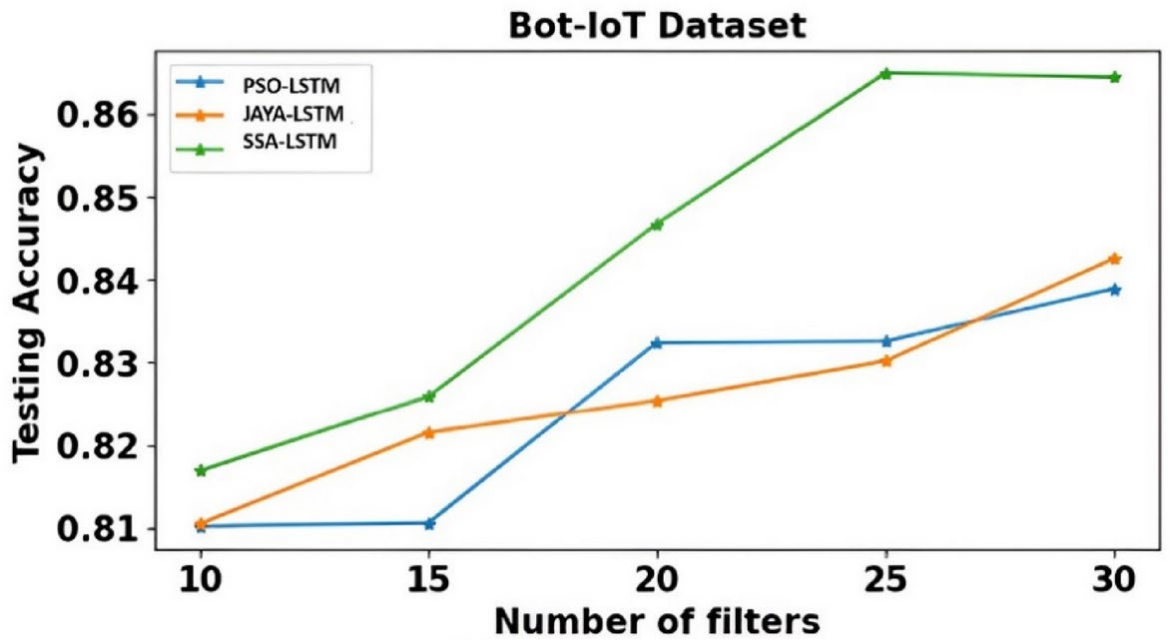
Testing accuracy comparison of PSO-LSTMIDS, JAYA-LSTMIDS, SSA-LSTMIDS on Bot-IoT dataset with SSA-LSTMIDS showing the highest accuracy.

Figure 6 illustrates the comparative analysis of testing accuracies across the NSL KDD dataset for the PSO-LSTMIDS, JAYA-LSTMIDS, and SSA-LSTMIDS models using filters between 10 and 30. Similarly, Figs. 7 and 8 present a comparative evaluation of testing accuracies among the same models on the CICIDS and Bot-IoT datasets. The findings suggest that the SSA-LSTMIDS model exhibited superior performance, particularly when configured with 30 filters. Notably, the most notable testing accuracy was attained using the CICIDS 2017 dataset, achieving a commendable accuracy rate of 99.80%.

**Fig. 9:**
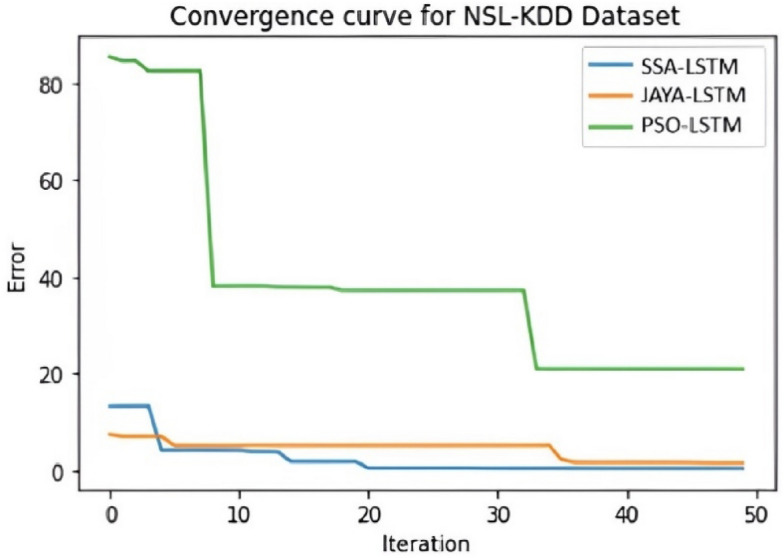
Convergence curve for NSL-KDD Dataset" illustrates error reduction over 50 iterations for three LSTM models: SSA-LSTM (blue line), JAYA-LSTM (orange line), and PSO-LSTM (green line). The y-axis shows error, and the x-axis shows iterations. SSA-LSTM quickly reduces error and converges near zero, JAYA-LSTM starts with slightly higher error but also converges near zero, while PSO-LSTM begins with a high error and gradually reduces, converging at a higher error level than the other two models. SSA-LSTM and JAYA-LSTM converge faster and to lower error values than PSO-LSTM.

**Fig. 10:**
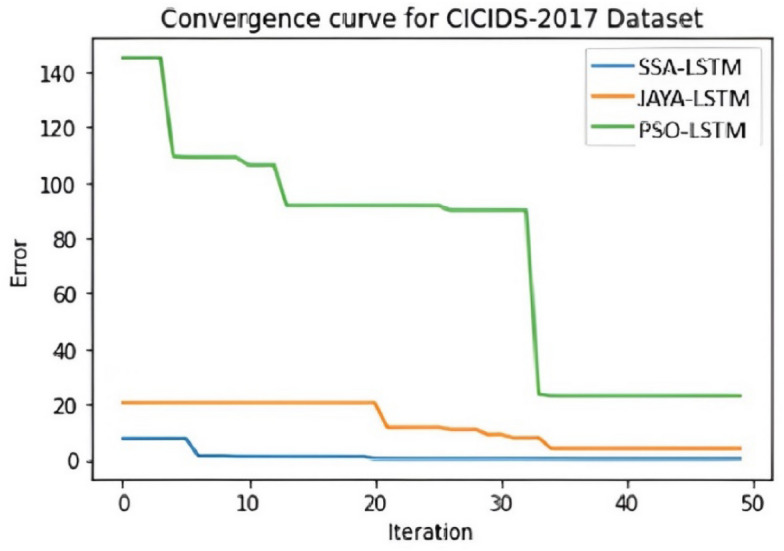
Convergence curve for CICIDS2017 Dataset shows error reduction over 50 iterations for SSA-LSTMIDS, JAYA-LSTMIDS, and PSO-LSTMIDS. SSA-LSTM and JAYA-LSTM quickly converge near zero, while PSO-LSTM reduces error gradually and converges at a higher level. SSA-LSTMIDS outperform JAYA-LSTMIDS and PSO-LSTM in error reduction speed and final error value.

**Fig. 11:**
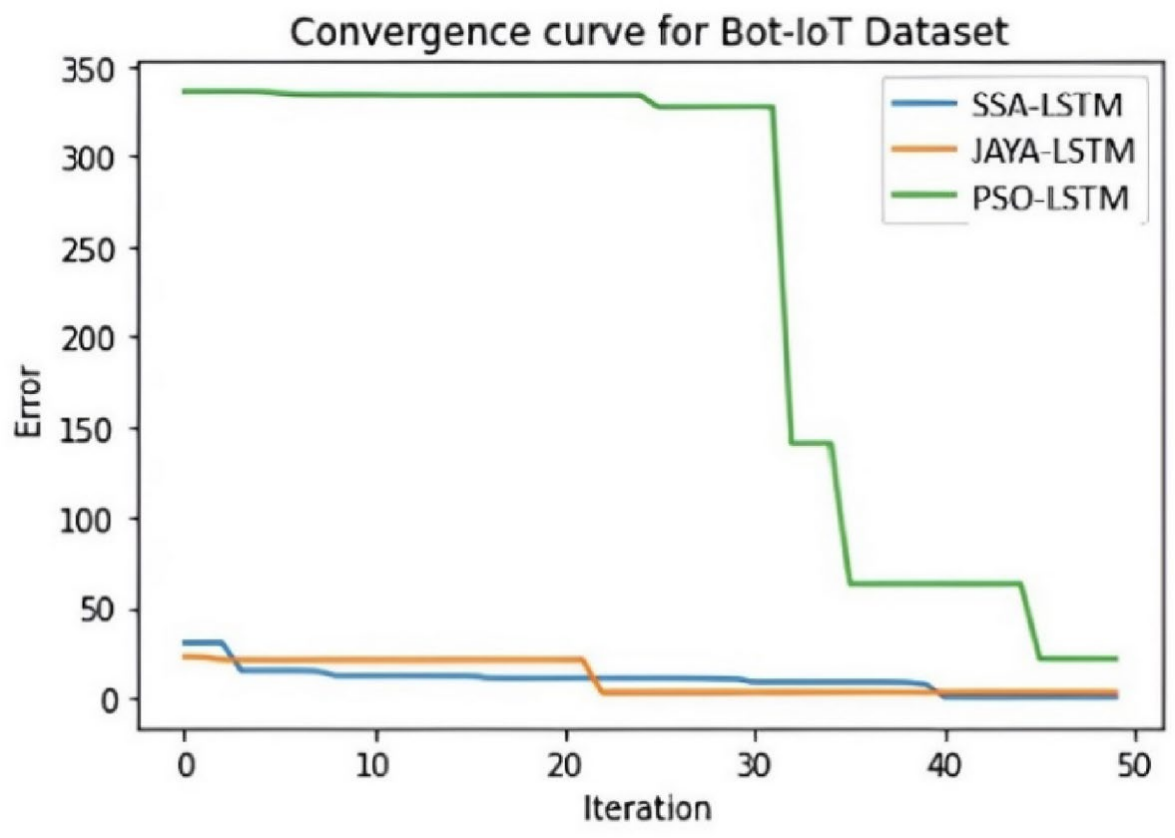
Convergence curve for BoT-IoT Dataset shows SSA-LSTMIDS and JAYA-LSTMIDS quickly converging to near-zero errors, while PSO-LSTMIDS converges slower and at a higher error level.

Convergence curve graphs in IDS classification are crucial for monitoring and optimizing the performance of intrusion detection models. Figures 9, 10 and 11 illustrates the visual representation of Convergence curve that demonstrates the relationship between the number of iterations (X-axis) and the classification error (Y-axis) during the training of an IDS model. The goal is for the curve to converge to a low value ie low error on the Y-axis, which suggests that the model has learned to make accurate classifications. When comparing the convergence curves of SSA-LSTM, JAYA-LSTM, and PSO-LSTM across NSL-KDD, CICIDS-2017, and Bot-IoT datasets, a consistent trend emerges. SSA-LSTM and JAYA-LSTM exhibit rapid convergence to lower error rates, outperforming PSO-LSTM, which displays slower convergence and higher error rates. Figure 9 highlights SSA-LSTM’s and JAYA-LSTM’s efficiency on NSL-KDD, while Fig. 10 showcases SSA-LSTM’s dominance on CICIDS-2017. In Fig. 11, SSA-LSTM excels yet again on the Bot-IoT dataset. These findings underscore the superior performance of SSA-LSTM in achieving quicker convergence to minimal error rates compared to PSO-LSTM and JAYA-LSTM across all the three datasets.

**Fig. 12:**
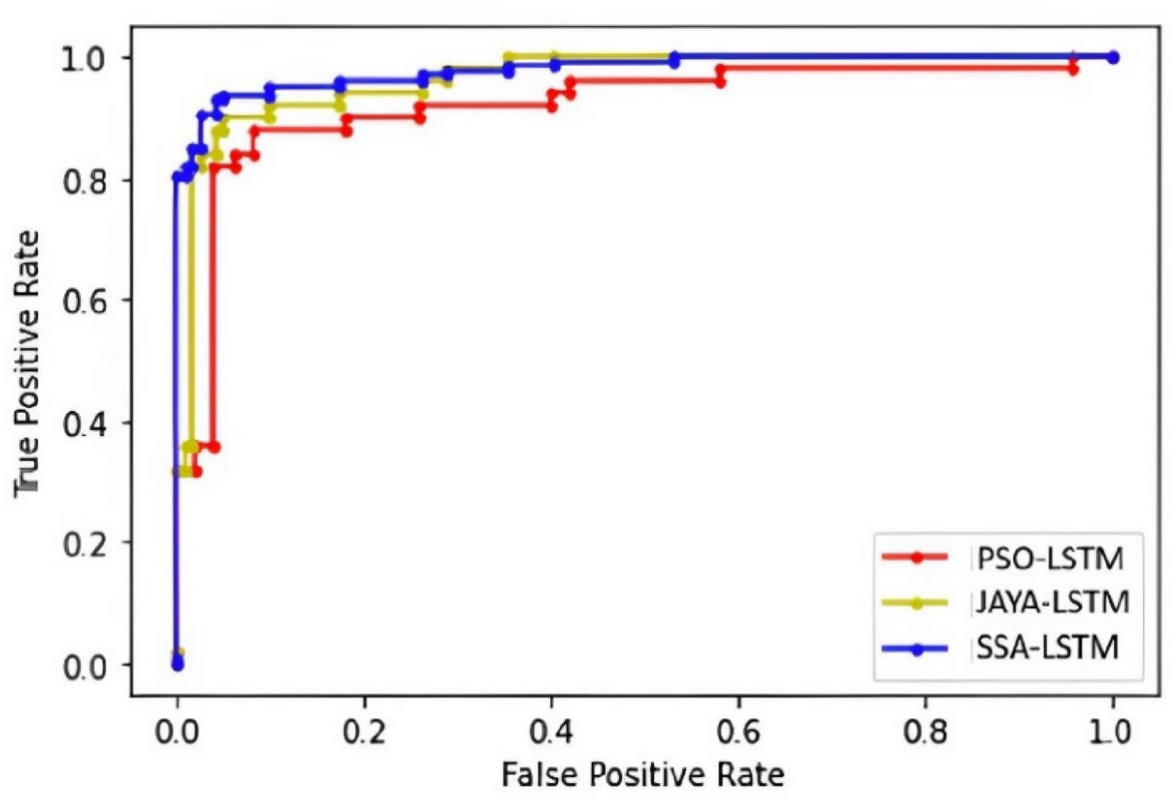
ROC curve showcases the performance of three LSTM models: PSO-LSTMIDS (red line), JAYA-LSTMIDS (yellow line), and SSA-LSTMIDS (blue line). The x-axis denotes the False Positive Rate (FPR), while the y-axis represents the True Positive Rate (TPR). PSO-LSTMIDS demonstrates commendable performance but lags marginally behind the others. JAYA-LSTMIDS surpasses PSO-LSTMIDS, closely rivalling SSA-LSTMIDS. SSA-LSTMIDS achieves the pinnacle of performance, approaching the top-left corner, indicative of superior true positive rates and minimal false positives.

Balancing TPR and FPR is critical in IDS classification. A high TPR is desired to ensure that actual intrusions are detected, but a high FPR can lead to too many false alarms, which can be costly and disruptive. Therefore, our model aims to strike a balance between these two rates by fine-tunning learning techniques to optimize TPR and FPR to meet the specific security and operational requirements of the environment. Figure 12 provides a graphical representation of Receiver Operating Characteristic (ROC) curve comparing three models: PSO-LSTM, JAYA-LSTM, and SSA-LSTM. The ROC curve plots the True Positive Rate (y-axis) against the False Positive Rate (x-axis) for different threshold values. All three models show high performance with curves close to the top-left corner, indicating a high True Positive Rate and a low False Positive Rate. SSA-LSTM appears to perform slightly better than PSO-LSTM, JAYA-LSTM, as its curve is consistently higher, indicating better classification accuracy.

The Optimized LSTM model, enhanced with cutting-edge optimization algorithms like PSO, JAYA, and SSA, demonstrates a remarkable balance between computational efficiency and performance in intrusion detection scenarios. This model’s computational complexity is notably lower compared to traditional deep learning approaches, making it particularly well-suited for real-time applications. The optimization techniques employed contribute to faster convergence and more efficient parameter tuning, reducing the overall computational burden. This lower complexity is a significant advantage in practical deployment scenarios, where rapid detection and response times are crucial for effective network security.

In real-time intrusion detection, where split-second decisions are paramount, the optimized LSTM model excels. Its streamlined architecture, coupled with the intelligent parameter optimization provided by PSO, JAYA, and SSA, allows for quick processing of incoming network traffic data. This efficiency doesn’t come at the cost of accuracy; rather, the model maintains high detection rates while operating with reduced computational overhead. The ability to swiftly analyze and classify potential threats in real-time is a game-changer for network security systems, enabling immediate responses to emerging cyber threats.

The model’s lower complexity also translates to broader applicability across various hardware configurations. This versatility is particularly valuable in diverse network environments, from large-scale enterprise systems to more resource-constrained IoT networks. By optimizing resource utilization, the model can be effectively deployed on a wider range of devices, enhancing the overall security posture of interconnected systems.

As shown below in Table 9 below, the effectiveness of the proposed model has been confirmed by comparing it with other relevant papers on different datasets and techniques.

**Table 9:** Performance comparison of the proposed model with other articles.

Article	Year	Model	Dataset	Accuracy	Precision	Recall	F-Score
Tang et al.^15^	2016	Deep Learning	NSL KDD	75.75	83	76	75
Kim et al.^16^	2016	LSTM-RNN	KDD Cup 1999	96.93	–	–	–
Ma et al.^17^	2016	Spectral clustering combined with Deep neural network (SCDNN)	KDD CUP 1999 & NSL KDD	92.03	–	91.35	–
Kim et al.^18^	2017	LSTM RNN	KDD Cup 1999	97.54	97.69		
Yin et al.^19^	2017	RNN-IDS	NSL KDD	83.28	–	–	–
Fu et al.^20^	2018	LSTM RNN	NSL KDD	97.52	–	–	–
He et al.^21^	2019	Multimodal-sequential approach with deep hierarchical progressive network (MR-DHPN)	NSL KDD	85.9	94.9	79.9	86.8
			UNSW-NB15	97	96.8	95.1	99.3
Wu et al.^22^	2019	LuNet employs LSTM for temporal features and CNN to learn spatial features	NSL KDD	99.05	–	–	-
			UNSW-NB15	84.98	–	–	–
Hassan et al.^23^	2020	Weight-dropped LSTM (WDLSTM) network	UNSW-NB15	97.1	–	–	98
Abbas et al.^24^	2021	Ensemble model integrating LR, NB, and DT with voting classifier	CICIDS 2017	88.96%	–	–	–
Ravi et al.^25^	2022	KPCA for dimensionality reduction, Utilizes RN and SVM for initial predictions, which are then stacked and fed into logistic regression	KDD Cup 1999	89	42	50	35
			UNSW-NB15	99	95	87	90
			WSN-DS	98	91	84	87
			CICIDS 2017	98	96	97	96
Zivkovic et al.^26^	2022	XGBoost classifier optimised with improved firefly algorithm	UNSW-NB15	91.42	81.67	98.84	88.91
Majhi et al.^28^	2023	Optimizing LightGBM using GOA	UNSW-NB15	97%	–	–	–
Kahtani et al.^29^	2023	PSO-GA for feature selection, GRU-LSTM for classification	CICIDS 2017	98.86	–	–	–
Donkol^30^	2023	ELSTM-RNN	UNSW-NB15	98.89	98	98	98
			CICIDS 2017	96.89	99.93	96.99	98.44
Li et al.^31^	2024	DT, RF, KNN, NB, MLP with feature selection & extraction techniques. RF with feature selection yields highest accuracy	ToN-IoT	88.22	86.99	89.6	87.69
Proposed model	2024	PSO-LSTM	NSL KDD	91.05	85.33	93.33	90.71
			CICIDS 2017	94.69	98.46	86.67	83.87
			BoT-IoT	83.89	76.97	84.63	80
		JAYA-LSTM	NSL KDD	95.14	90.68	1	95.3
			CICIDS 2017	97.14	99.17	94.31	92.61
			BoT-IoT	84.25	78.57	88.18	82.29
		SSA-LSTM	NSL KDD	97.89	91.67	1.00	97.30
			CICIDS 2017	99.80	1.00	94.44	94.44
			BoT-IoT	86.44	80.21	89.28	85.51

**Fig. 13:**
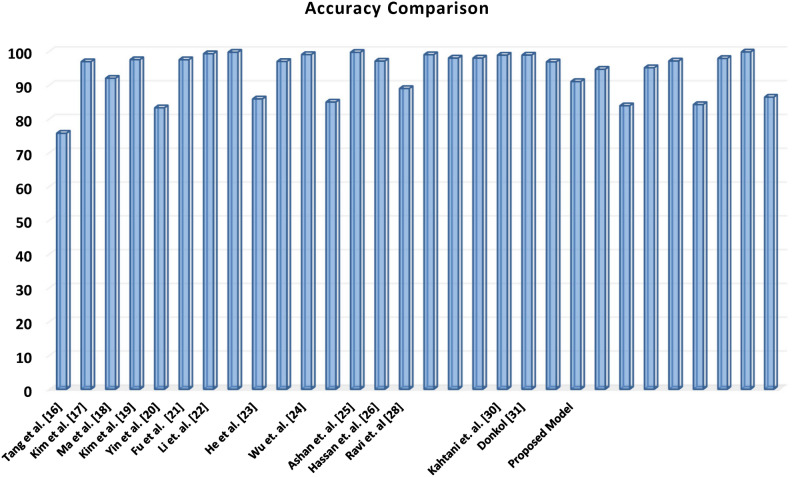
Accuracy Comparison is a bar chart evaluating the accuracy of different models, including the proposed model, against several other studies. The x-axis lists the various authors and their respective studies, while the y-axis indicates accuracy, scaling from 0 to 100. The height of each bar reflects the accuracy accomplished by the corresponding model. Notably, the proposed model exhibits the highest accuracy among all the compared studies, underscoring its superior performance.

Assessing the accuracy of the proposed model involves evaluating its performance in accurately classifying data when compared to models discussed in other research articles. These comparisons offer insights into the effectiveness and competitiveness of the proposed model relative to existing approaches within the field as demonstrated in Fig. 13.

## Conclusion and future scope

The paper introduces an optimized LSTM-based Intrusion Detection System (IDS) model designed to effectively classify normal and malicious network traffic. The experimentation in this study uses NSL KDD, CICIDS, and Bot-IoT dataset, for analysis and evaluation. PSO, JAYA and SSA optimization techniques are implemented to fine tune the hyperparameters of LSTMIDS model. The efficiency of the proposed IDS has been validated using various performance evaluation metrics including training and testing accuracy, precision, recall, F-score, and ROC. Comparative analysis of the optimization techniques on the three datasets are carried out. Experimental results reveal that SSA-LSTMIDS model yields better performance as compared to PSO-LSTMIDS and JAYA-LSTMIDS based models. SSA-LSTMIDS demonstrates superior performance compared to the other two models, as evidenced by higher classification accuracy, lower false alarm rates, and superior performance across various metrics examined in this study. Hence, the proposed model proves to be highly effective for real-time intrusion detection applications. However, in this paper, we tested with only one type of LSTM. In future studies we will explore additional deep learning algorithms, multiple variants of LSTM along with evolutionary techniques for optimization to capture intricate patterns in network traffic.

## Data Availability

The datasets used and/or analysed during the current study are available from the corresponding author on reasonable request.
